# Evaluation of Community-Based Dog Welfare and Rabies Project in Sanur, a Sub-district of the Indonesian Island Province of Bali

**DOI:** 10.3389/fvets.2019.00193

**Published:** 2019-07-09

**Authors:** Ni Wayan Arya Utami, Kadek Karang Agustina, Kathryn Nattrass Atema, Gusti Ngurah Bagus, Janice Girardi, Mike Harfoot, Yacinta Haryono, Lex Hiby, Hendra Irawan, Pande Putu Januraga, Levin Kalalo, Sang Gede Purnama, I. Made Subrata, Ida Bagus Ngurah Swacita, I. Made Indrayadnya Swarayana, Dewa Nyoman Wirawan, Elly Hiby

**Affiliations:** ^1^Department of Public Health and Preventive Medicine, Faculty of Medicine, Udayana University, Denpasar, Indonesia; ^2^Department of Veterinary Public Health, Faculty of Veterinary Medicine, Udayana University, Denpasar, Indonesia; ^3^International Fund for Animal Welfare, Yarmouth Port, MA, United States; ^4^Bali Animal Welfare Association (BAWA), Ubud, Indonesia; ^5^Independent Researcher, Cambridge, United Kingdom; ^6^Conservation Research Ltd., Cambridge, United Kingdom

**Keywords:** dog, canine, rabies, vaccination, animal welfare, community engagement, Bali

## Abstract

The Indonesian island province of Bali experienced its first rabies incursion in 2008. Mass vaccination of the dog population has proven effective and rabies cases in dogs and people have decreased, however the virus is still circulating among the dog population. Vaccination coverage must be maintained until rabies elimination. Increasing efficiency and effectiveness of vaccination campaigns is therefore desired. Community engagement leading to preventative health actions by community members can reduce disease incidence and costs of control. Here we evaluate 2 years of a novel community-based dog welfare and rabies control project (Program Dharma) in the Sanur sub-district. The project engaged the services of people living in the project area with an interest or experience in dogs or community health services. These people spoke with owners within their own community about dog welfare and health, monitored owned and unowned dogs and increased owner and carer efforts to access vaccination and further veterinary services. The evaluation focused on a sample of dogs whose owners had been regularly engaged with project. Vaccination coverage was increased and there were no dog or human rabies cases reported in the project area; the percentage of the dogs that had never been vaccinated was reduced by an average 28.3% (baseline unvaccinated 41–49%, post-project unvaccinated 11–19%). The welfare of dogs improved from an average of 20.7% of dogs with visible welfare problems at baseline to 2.7% after project implementation. Roaming dog density observed on street surveys also decreased in all project areas (24–47% reduction dependent on *desa*). A participatory evaluation event with a sample of Program Dharma community-based agents highlighted several additional successes, including that the community appeared to welcome and value their services and were beginning to support the cost of project activities. Conversely, challenges included identifying dogs in the database during revisits, sustaining the costs of community member time spent working on Program Dharma activities and the costs of veterinary care, whilst avoiding dependency of owners on free veterinary services. The benefits revealed by the evaluation were judged to be sufficient to extend Program Dharma to new areas, whilst evolving activities to resolve challenges.

## Introduction

Rabies has spread throughout Indonesia and is maintained in the domestic dog population in the majority of the 33 Indonesian provinces (1). The Indonesian island province of Bali was historically rabies-free until an incursion in 2008 (2). A combination of culling unconfined dogs with strychnine and vaccination of dogs at central point locations using locally manufactured vaccine failed to contain the outbreak; by 2010, rabies cases had been confirmed in all nine regencies in Bali (2, 3). Following concerted efforts by local, national and international agencies, the first island-wide mass dog vaccination campaign was launched in October 2010 (2). Several mass dog rabies vaccination drives were run in subsequent years, with resulting decreases in the number of both dog and human rabies cases of the disease (4); based on government records, 476,459 dogs were vaccinated in 2018 (5). Although human rabies cases are decreasing, the rabies virus is still present in Bali. In 2017, 15,630 people received post-exposure prophylaxis following dog bites, there were 93 laboratory confirmed dog rabies cases and two people are confirmed to have died of rabies (5, 6).

Mass vaccination of the dog population can lead to herd immunity and hence prevention of rabies virus spread and eventual elimination of infection in dogs and therefore people (7, 8). For vaccination to be effective and efficient in reducing both dog and human rabies, at least 70% of the dog population must be immunized during annual campaigns (9). Vaccination coverage must also be comprehensive, leaving no pockets of unvaccinated dogs (3). Maintenance of effective vaccination coverage is challenged by high population turnover resulting from births of susceptible puppies and deaths of vaccinated dogs (7), and the import of unvaccinated dogs. Studies of dog demography and vaccination in Bali suggest similar challenges to reaching and maintaining vaccination. Including failure to vaccinate puppies, roaming and abandonment of owned dogs making them less accessible for vaccination and high population turnover between campaigns (4, 10). Although data to test this hypothesis is not currently accessible in Bali, we note further pulses in population turnover and import of unvaccinated dogs could be triggered by sporadic culling.

Although the strategy of mass dog vaccination has proved to be successful in many countries (8, 11–14), and is the accepted policy for rabies control in Indonesia, sustaining the costs of vertically structured programs is challenging. Community engagement has been found to lead to preventative health actions by community members, effectively reducing incidence of disease and the associated cost of vertical control for governments (15–18). We therefore proposed a similar approach in the form of community-based dog welfare programs as a solution for achieving efficient and sustainable rabies control. The aim of this study was to evaluate the impact of involving community members as influencers of their local dog owners' behavior to maintain herd immunity of dogs by improving dog welfare and inspiring dog owners to provide better care their dogs. Following 2 years of implementation of this novel community-based approach, we conducted a participatory evaluation with focus on exploring lessons learnt combined with quantitative data analysis, the results of which are reported here.

## Materials and Methods

### Study Area

Three neighboring *desas* (villages) in the Sanur area of Bali were selected for the intervention: Sanur Kaja, Sanur Kauh and Kelurahan Sanur, with a total human population of 24,373 (local government statistics reported as a pers. comm. from government representative) and estimated dog population of 6,009 dogs (10). Each *desa* was comprised of between eight and 11 *banjars* (sub-villages). These *desas* had experienced their last human rabies death in 2010 and last reported dog rabies case in April 2012, but remained vulnerable to rabies as the virus continues to be reported in other areas of Bali. Rabies surveillance is supported by the use of integrated bite case management where any suspect bite case entering a health facility is reported for follow-up by a government livestock officer, to assess the biting animal for clinical symptoms. Brain samples of suspect rabid dogs are sent for examination using Direct Fluorescent Antibody Testing at the Disease Investigation Center (Balai Besar Veteriner) (19).

### Program Dharma Intervention

**Figure 1:**
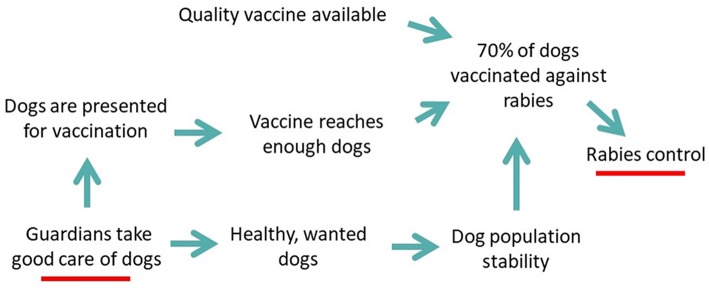
Illustration of the theory of change for Program Dharma contribution to rabies control via improvement in dog care practices.

Program Dharma was designed around a theory of change (Figure 1) to control rabies via community-led improvements in dog care practices; the program was primarily implemented by trained community members (T2s, see Table 1), conventionally termed “community-based agents.” These T2s were selected by *desa* leaders because they had an interest or experience in dogs or community health services and lived in the same *desa* as the dog owners they would be expected to influence. Each T2 was responsible for one or two banjars; banjars vary in size and so smaller banjars were paired up. The T2s volunteered for approximately 10 h/week for Program Dharma for which they received a small financial incentive. Their primary responsibilities were to conduct informative and informal visits with dog owners and other locals during which they (i) assessed the welfare state of dogs (visual appraisal of dog body condition, presence of skin problems and/or injury—see 10), (ii) monitored dog care practices and (iii) maintained a digital record of all dogs in their *banjar*. They were also responsible for helping to facilitate Program Dharma social and educational activities in their *desa*, including “Health Days” where the community could access free preventative veterinary care for dogs, provided by the Bali Animal Welfare Association (BAWA) and local government livestock officers, including sterilization (surgical spay or neuter), rabies vaccination and de-worming. In addition, T2s were actively involved in “troubleshooting” dog-related concerns from locals, including encouraging dog bite treatment according to health department advice, promoting the use of an emergency hotline set up to handle emergency calls by the Bali Animal Welfare Association, facilitating within-*banjar* adoptions of unwanted dogs and puppies, and maintaining positive relationships with *desa* leadership. Dog owners were not expected to pay for Program Dharma services or for rabies vaccination accessed through government run annual vaccination campaigns.

**Table 1:** Responsibilities of the two key roles in Program Dharma; the mentor (T1) and the community-based agent (T2).

**Program Dharma mentor (T1) *N* = 7** Train T2s in basic animal welfare and door-to-door owner socialization Managing, motivating and evaluating T2s Support T2s in baseline data collection Help T2s plan regular community events and fundraising activities Facilitate coordination with local veterinary service providers Oversee *desa*-level activities Project reporting and communication	**Program Dharma community-based agent (T2) *N* = 22 (Sanur Kaja *N* = 4, Kelurahan Sanur *N* = 7, Sanur Kauh *N* = 11)** Through door-to-door socialization with owners and regular street surveys, document each dog in *banjar* and monitor welfare and rabies vaccination status Organize regular access to veterinary services for *banjar* dogs Regular meeting with T1s and other T2s to assist in the organization of community events, small group meetings and public education. First point of contact for any dog problems in *banjar*

The T2s were trained and managed by mentors, mostly recent graduates of Udayana University with degrees in Veterinary or Public Health, termed T1s (see Table 1). In addition, T1s provided additional support in local event organization, project communications and staffed the emergency hotline. Each T1 spent an average of 40 h/week on Program Dharma activities for which they received a salary. Each *desa* was assigned one T1 as village coordinator. T1s were trained over 6-weeks, receiving in-person sessions on animal behavior; health and welfare; rabies signs, symptoms and prevention; community engagement strategies (e.g., door-to-door animal welfare consultations and mass communication using social media and hard copy formats such as banners and booklets); leading, managing and mentoring; and data collection and monitoring strategies. The sessions were designed and delivered through collaboration between BAWA, Udayana University and the International Fund for Animal Welfare (ifaw).

Five Udayana University field coordinators and staff from BAWA provided additional oversight to the T1s, and they were also responsible for liaising with local government departments including the Dinas for Animal Health-Denpasar to coordinate government-sponsored rabies vaccination activities and *desa* leadership to facilitate ongoing project support. The field coordinators spent between 1–10 h/week on Program Dharma activities. Program Dharma received support from ifaw who provided guidance and adaptive management, data analysis and periodic evaluation.

A theory of change for the how the Program Dharma intervention contributed to rabies control is provided in Figure 1. Table 1 provides a summary of key roles and responsibilities of T1s and T2s.

#### Intervention Activities

From launch in May 2016 to June 2018, Program Dharma completed 621 dog sterilizations (unowned dogs prioritized for sterilization) during the 74 “Health Days” across all three *desas*. Program Dharma also supported the local veterinary authorities in accessing dogs for their government funded rabies vaccination leading to 3103 vaccinations in 2016, 3,576 in 2017 and 3,381 in 2018.

### Data Collection

#### Dog Demography, Health and Welfare Status

Baseline survey data was collected for each dog during the first visit from Program Dharma starting in May 2016. Data was collected using a mobile phone application as described in Hibyet al. (10). This survey was designed in two parts (see Hiby et al. (10), Supplementary Materials for the survey questionnaires): (1) The “Dogalog” recorded permanent details about the dog, such as its sex, breed type and date of birth; (2) The “DogStatus” recorded transient details on the day the dog was seen by the Program Dharma team including its body condition score (five point scale from emaciated through to obese; body condition score training tool available at https://www.icam-coalition.org/project/indicators-project/), presence of a visible skin problem, presence of visible wounds, rabies vaccination status, breeding status and confinement. A dog was categorized as in “poor welfare” if observed to be suffering from any of the following conditions: body condition score of emaciated or thin, visible skin problem or a visible wound. Confinement was based on the owners report of the method of confinement used for the majority of the dog's average day, including house and/or yard (defined as an outdoor space with some kind of physical boundary, usually a wall), kennel or cage, tether or chain and free to roam. Whether this method of confinement was effective, e.g., whether the yard boundary was truly dog-proof, was not investigated during the visit. Further details of the methods used to score transient details are also described in Hiby et al. (10). Data were collected during repeated visits to create a history of the health and welfare of each dog included in Program Dharma.

The data was recorded using either of two smart phone apps: Epi Info™^1^ (a database and statistics program for public health professionals, developed by the Centers for Disease Control CDC, Atlanta, GA, USA) or Device Magic^2^ (a mobile phone application for data collection), in combination with Wise Monkey^3^ (a web-based database and data management system that works in tandem with mobile phone applications, developed by the Wise Monkey Foundation).

A sample of 2,098 dogs were included for analysis, these were all the dogs that had received at least two visits from a Program Dharma team member and where the most recent visit had occurred in the previous 12 months (between June 2017 and June 2018). Most dogs received two visits (*n* = 1,392, 66.3%), 277 (13.2%) received three visits, 272 (13.0%) received four visits, with decreasing frequency until only one dog received the maximum of eight visits. This sample compares the population of dogs at baseline with the same dogs at their most recent revisit—thus testing the hypothesis that being part of Program Dharma improves dog welfare and vaccination status. This sample represents 35% of the 6,009 dogs found during the census baseline conducted between May 2016 and May 2017 (10). We excluded data from 3911 dogs (65%), because they had either not been revisited at all since the census (*n* = 2,705, 45%; usually because they could not be tracked down) or their most recent revisit had been >12 months previously (*n* = 1,206, 20%).

##### Statistical analysis

The McNemar statistical test looks at repeated samples; where one subject is acting as its own control in a “before” and “after” scenario. We compared the rabies vaccination status of each dog at baseline, before Program Dharma was active in the *desa*, to the vaccination status at the most recent revisit by a Program Dharma team member. However, McNemar test can only be used with dichotomous variables, so a current vaccination status was compared to a combined status of unvaccinated, including both never vaccinated and where vaccination had lapsed beyond 12 months. McNemar tests were also used to explore physical welfare for each dog at baseline compared to the most recent Program Dharma visit.

#### Roaming Dog Density

A street survey using direct observation of roaming dogs on set routes through public streets was conducted in all *banjars*. The routes were designed to cover as many of the streets as possible within a *banjar* but following an efficient route that minimized retracing previously surveyed streets. Surveyors worked in pairs, traveling on motorbikes at an average of 10 km per hour, with the passenger observing and recording the presence of roaming dogs using the smart phone app OSMTracker. Using routes with minimal retracting of steps, and motorbikes for efficient progress along routes, avoids the risk of double counting dogs. This method is described in more detail in Hiby et al. (10). The same survey protocol was followed for the same set of routes every 6–8 months providing a measure of density of roaming dogs per km of street surveyed over time. Density expressed as dogs seen per km predicts the number of dogs people will encounter as they travel along public streets and is therefore more meaningful than estimates expressed as total abundance or dogs per unit of area such as km^2^ (20). Roaming dog density was measured over the following time periods: Kelurahan Sanur July 2016—December 2016; Sanur Kaja July 2016–September 2017; Sanur Kauh June 2016–February 2018. The extent of the change in density (dogs per km of street surveyed) over time (year) as a continuous variable for each *desa* was tested using Analysis of Covariance, including dummy variables to control for the difference in density between *banjars*. Because we expected an exponential decay in density over time, as opposed to a linear change, the log of dog density was used as the dependent variable in the ANCOVA. The resulting coefficient for time was back transformed to give the percentage change in density.

#### Perceptions of Successes and Challenges

In addition to the data analysis described previously, a series of onsite participatory exercises were carried out over 3 days in April 2018 to establish the perceptions of successful change and challenge by the Program Dharma team, including T2s. Two days were predominately planning and prioritization of lessons learned by the core management team. One day involved 30 participants (16 men and 14 women), including nearly all of the T2s. The T2s mostly worked through the participatory exercises in *desa* teams facilitated by their three T1 village coordinators. There were also some plenary discussions led by a member of the Program Dharma management team who was experienced at facilitation. These plenary sessions included prioritization exercises to identify successes and challenges that participants felt were most important to them and the communities they were working in. This participatory evaluation event was conducted primarily in Indonesian. Facilitation notes and participatory exercises are provided as Supplementary Materials.

## Results

### Dog Demography, Health and Welfare Status

#### Dog Demography

**Figure 2:**
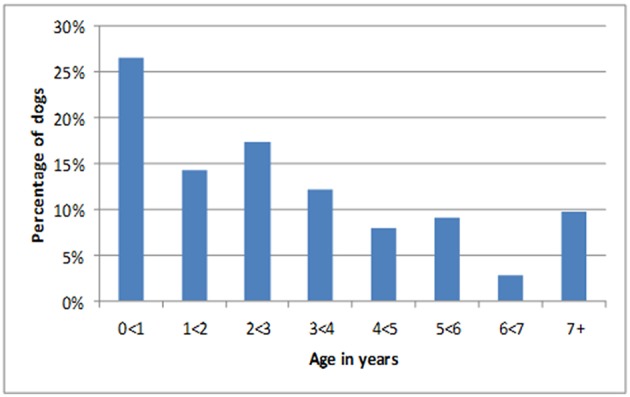
Age distribution for all dogs at baseline.

The 2098 dogs lived in three *desas*; Sanur Kaja (*n* = 519), Kelurahan Sanur (*n* = 761) and Sanur Kauh (*n* = 818). Almost all dog-owning households gave consent to be interviewed at first request, when concerns were raised, a repeat visit accompanied by representatives from *desa* leadership was usually sufficient to reassure the dog owner of project legitimacy. Only 3 households were recorded to continue to refuse participation. The 2,098 dogs were comprised of 22.6% “Bali dogs,” a recognized breed native to Bali, 44.8% were identifiable as other breeds and 32.6% were mixed-breed dogs. There was a slight skew toward male dogs (53.5% male: 46.5% female) and the vast majority were owned dogs (98.3%) with a few unowned dogs (1.7%). Figure 2 shows the age distribution of the dogs at baseline, in which dogs under 1 year of age were the largest age category (*n* = 556, 26.5%). This population composition (in terms of age distribution, breed type, sex, source and confinement method) was not significantly different to the population reported at the baseline census, although the proportion of unowned dogs was higher at baseline (3.3%) (10). At the most recent revisit, 461 (22.0%) dogs were reported to have left the household, just over half of these had died (*n* = 240; 11.4%). The remaining had been given away to another household (*n* = 109; 5.2%), disappeared (*n* = 58; 2.8%) or been sold (*n* = 19; 0.9%). The time between baseline and revisit varied between dogs hence these fates of dogs that had left the household occurred over a timescale ranging from 13 to 757 days (average 443 days).

#### Rabies Vaccination Coverage

**Figure 3:**
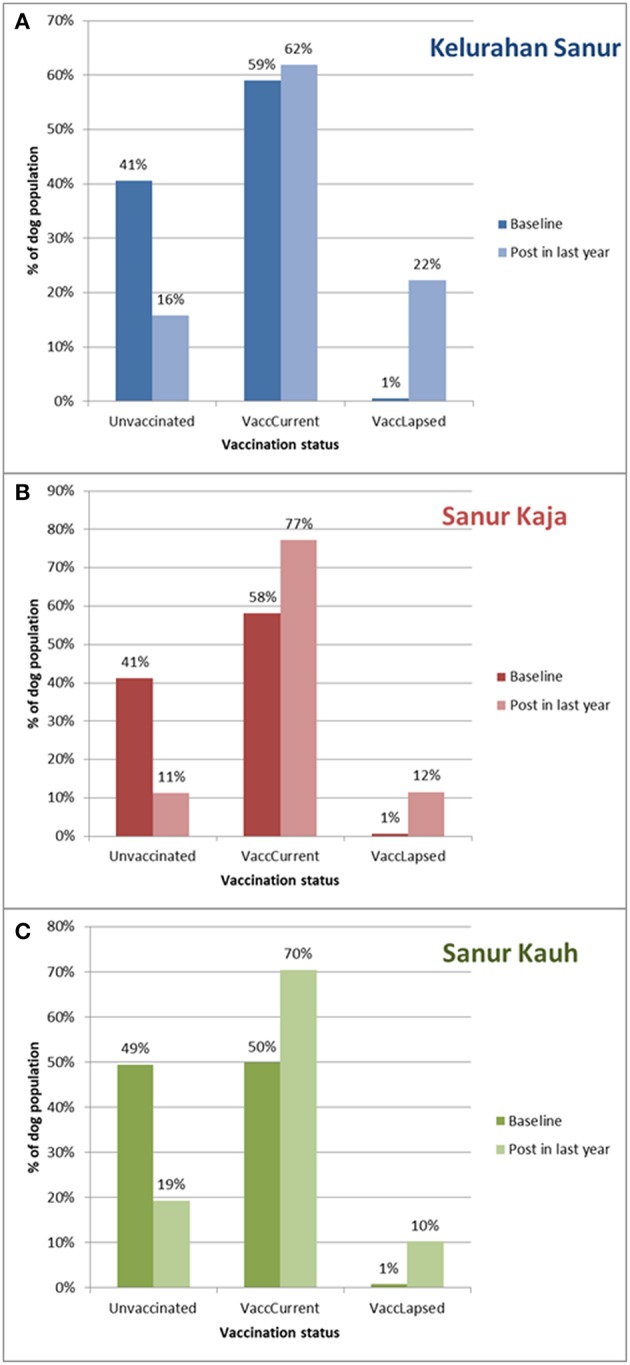
**(A–C)** The percentage of dogs unvaccinated (Unvaccinated), or with a current (VaccCurrent) or lapsed (VaccLapsed) rabies vaccination at baseline and post Program Dharma intervention for all three *desas* (Kelurahan Sanur, Sanur Kaja, Sanur Kauh). “Post in last year” refers to revisit occurring between June 2017 and June 2018. Date of “Baseline” differs for each dog according to when they joined the project, majority (*n* = 1952; 93%) were part of census conducted between May 2016 and May 2017 for project set-up. A further 146 (7%) dogs joined later as they moved into the project area or were born to local dogs.

All *desas* showed a decrease in the percentage of dogs that had never been vaccinated against rabies, from between 41–49% at baseline to between 11–19% at most recent visit; an average decline of 28.3% (Figures 3A–C). Although all *desas* also show an increase in the proportion of dogs that are rabies vaccinated, there is also an increase in dogs with lapsed rabies vaccination history; the category of lapsed vaccination is assigned when the date of the dog's most recent vaccination is over a year prior to the date of the visit by the T2. We find that Sanur Kauh (McNemar X^2^ = 21.393, *p* < 0.001) and Sanur Kaja (McNemar X^2^ = 22.785, *p* < 0.001) have shown an increase in the proportion of dogs with a current vaccination as compared to unvaccinated/lapsed. However, in Kelurahan Sanur (McNemar X^2^ = 0.00408, *p* = 0.949), although the proportion of dogs that had never been vaccinated decreased, the proportion with a lapsed vaccination increased, leading to a non-significant change in the proportion of dogs with a current vaccination status in that *desa*.

#### Physical Health

**Figure 4:**
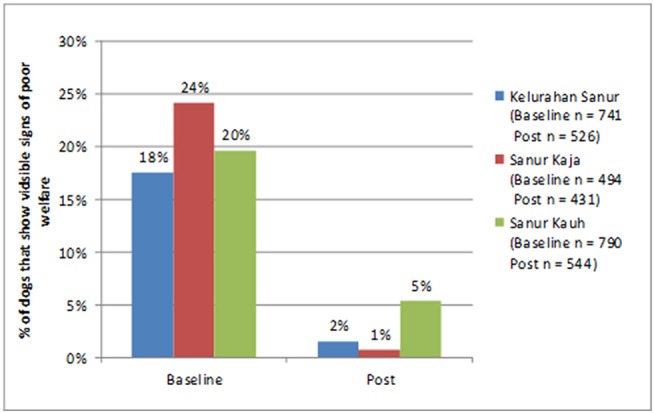
Change in the percentage of dogs in a state of poor visible welfare at baseline and at their most recent visit.

The percentage of dogs with poor physical welfare (defined as poor body condition and/or a visible skin problem and/or visible injury) was significantly reduced at the most recent revisit as compared to baseline in all three *desas* (Kelurahan Sanur: McNemar X^2^ = 69.063, *p* < 0.001, Sanur Kaja: McNemar X^2^ = 90.163, *p* < 0.001, Sanur Kauh: McNemar X^2^ = 41.953, *p* < 0.001). Figure 4 shows the extent of the reduction in the percentage of dogs in poor welfare.

#### Sterilization

**Figure 5:**
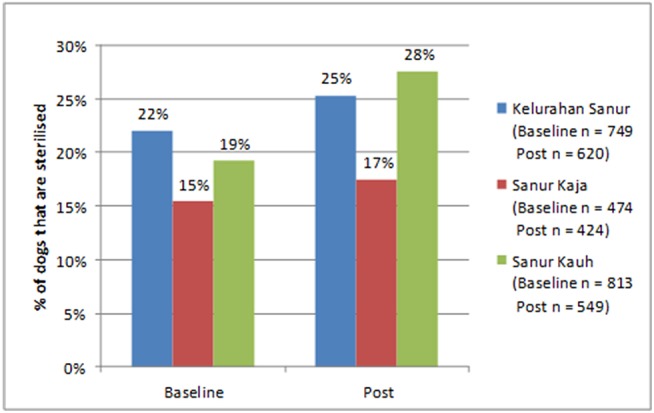
Change in percentage of dogs that are sterilized at baseline and at their most recent visit.

Sterilization of dogs was performed at Program Dharma “Health Days” and independently by private vets. There was an increase in the percentage of dogs that were sterilized in all three *desas*, see Figure 5; however, this was only statistically significant in Sanur Kauh (Kelurahan Sanur: McNemar X^2^ = 0.0488, *p* = 0.825, Sanur Kaja: McNemar X^2^ = 0.381, *p* = 0.537, Sanur Kauh: McNemar X^2^ = 7.682, *p* = 0.00558).

#### Confinement

**Figure 6:**
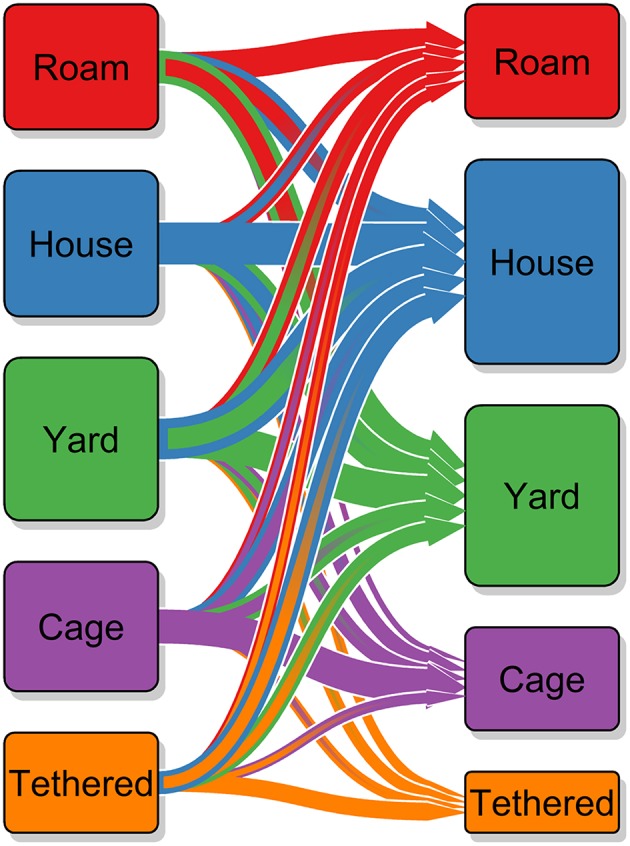
Sankey diagram of transition in dog confinement method used by owners at baseline and at the most recent visit. The arrows show the direction from baseline to most recent visit, the size of the arrows and the size of the cuboid indicate the number of dogs involved; confinement at baseline is shown by the cuboids on the left and confinement at last revisit on the right. The outline color of the arrows also indicates which confinement method the arrow will end up at; the fact that nearly all the largest arrows have a blue outline indicates that a move to being confined to the house is the most common transition.

Over the period of Program Dharma there was a change in confinement practices for dogs. In Table 2, the confinement data is presented as a transition matrix. The cells have been color coded according to welfare concern; the cells in green are dogs that were once kenneled/caged or tethered and are now confined to a house or yard (*n* = 165), potentially contributing to an improvement in their welfare. The cells in red are dogs that were once roaming or confined to a house or yard and are now reported to be kenneled/caged or tethered (*n* = 64), potentially a welfare concern caused by a move to extreme confinement. Figure 6 presents the movement of dogs between confinement methods in a Sankey diagram.

**Table 2:** Transition matrix of confinement practices for dogs scored at baseline and at their most recent visit.

		**Confinement at most recent revisit (revisited within last year)**					
		**Roam**	**House**	**Yard**	**Kennel/cage**	**Tethered**	**TOTAL**
Baseline confinement	Roam	**73**	85	93	2	0	253
	House	24	**155**	83	20	7	289
	Yard	42	145	**116**	24	11	338
	Kennel/cage	10	46	48	**100**	9	213
	Tethered	10	44	27	9	**26**	116
	TOTAL	159	475	367	155	53	1209

*Bold text highlights the numbers of dogs whose confinement did not change over time. The cells have been color coded according to welfare concern; the cells in green are dogs that were once kenneled/caged or tethered and are now confined to a house or yard. The cells in red are dogs that were once roaming or confined to a house or yard and are now reported to be kenneled/caged or tethered*.

### Roaming Dog Density

**Figure 7:**
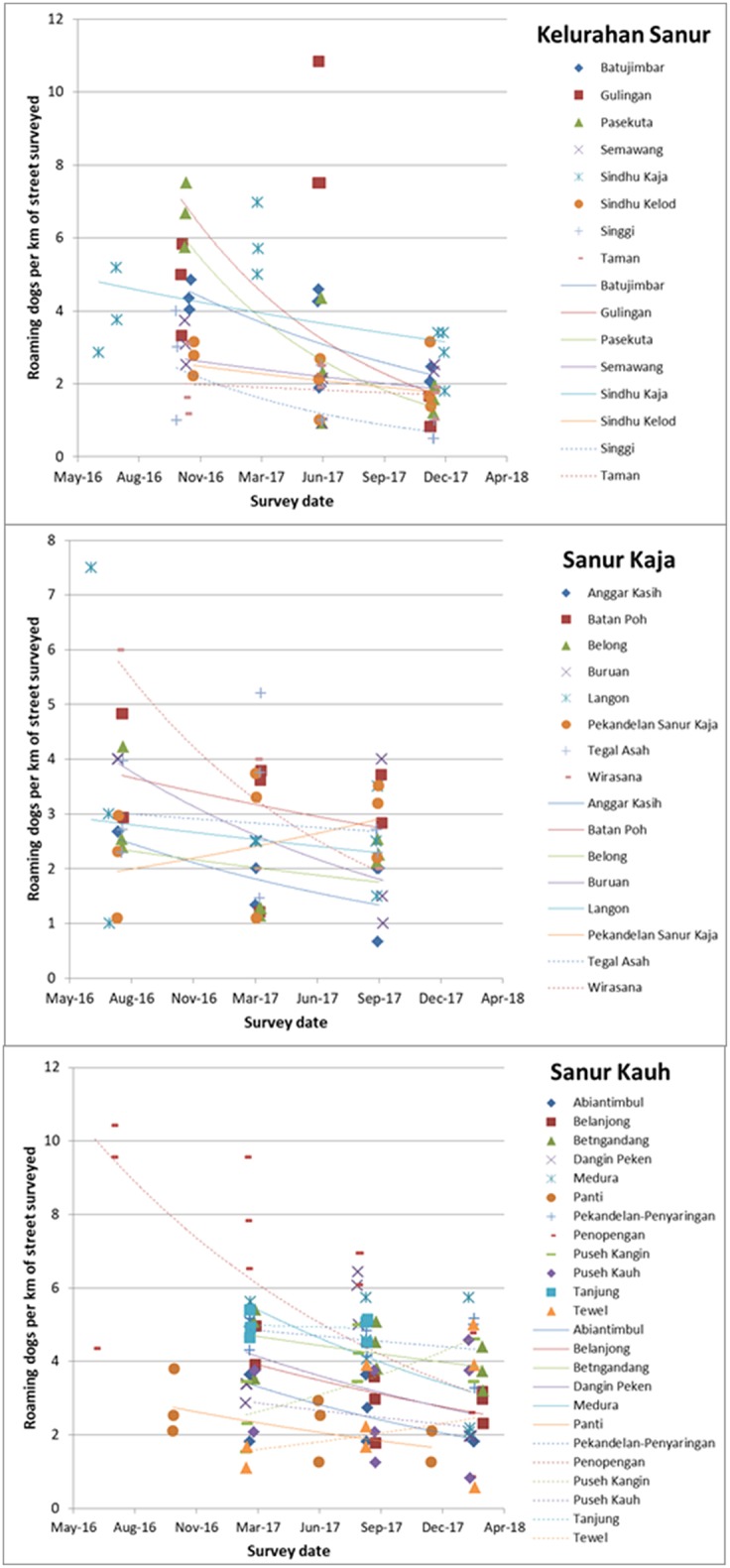
**(A–C)** Change over time in the number of roaming dogs observed per km of street surveyed for each *banjar* within the three *desas* (Kelurahan Sanur, Sanur Kaja, Sanur Kauh). Colored icons represent roaming dog density on each of three replicate surveys along the same *banjar* survey route, across three street survey events every 6–8 months, with best fit exponential trendlines indicating the trend over time for each *banjar*.

There has been a statistically significant decline in the density of roaming dogs in all three *desas* over time. In Kelurahan Sanur there was an average 47.2% reduction per year, equivalent to a reduction of 2.75 dogs per km of street surveyed in the first year (*F* = 7.448, *d.f*. = 8, *p* < 0.001); in Sanur Kaja there was an average 23.6% reduction per year, equivalent to a reduction of 0.51 dogs per km of street surveyed in the first year (*F* = 1.896, *d.f*. = 8, *p* = 0.0339); and in Sanur Kauh an average 27.7% reduction per year, equivalent to a reduction of 1.04 dogs per km of street surveyed in the first year (*F* = 5.228, *d.f*. = 12, *p* = 0.00207). The density observed in each *banjar* and individual best fit trendlines assuming an exponential decay over time is shown in Figures 7A–C. This illustrates that the rates of decay are similar in most banjars.

### Perceptions of Successes and Challenges

The Program Dharma team, including the T2s, used participatory exercises to interpret the results from the data analysis and to develop agreed statements about Program Dharma successes and challenges. These statements were scored in terms of perceived importance using a combination of plenary discussion and participatory voting exercises where each participant had an equal number of “votes” to select the successes that mattered most to them and their community. The following lists the priority successes and challenges according to this scoring.

Success statements:

1. More dogs are physically healthy / there are fewer skinny or skin problem dogs.
2. Owner knowledge of good dog care practices has increased.
3. There is an increased knowledge of rabies prevention, both rabies vaccination of dogs and effective treatment of dog bites, this results in fewer instances of rabies related panic in the public.
4. Program Dharma is an innovative program that makes Sanur *desas* inspirational to others
5. We have built an accessible and dynamic estimate of the dog population in terms of numbers and vaccination status.
6. There have been no dog or human rabies cases in any of the Program Dharma *desas*.
7. Vaccination services are delivered more regularly to the community and the community knows about how to access these services.
8. There are fewer roaming dogs on the streets.
9. Owners treat their dogs better; providing food, water, kindness and rely less on caging and tethering.

Challenges:

1. The cost of providing veterinary care through health days is high and currently covered by the Bali Animal Welfare Association, this is unsustainable in the long term.
2. Dog owners may be learning to depend on the free veterinary services provided by Program Dharma.
3. Accessing local sources of funding to support the costs of Program Dharma is time consuming.
4. The veterinary authorities will not allow Program Dharma vets to conduct rabies vaccination themselves, this must be done by government vets; these government vets are busy with many other roles and so vaccination services are delayed, vaccination coverage hence lower than it could be with Program Dharma vets allowed to vaccinate as well.
5. Program Dharma has a social media presence that helps build awareness of the project and can be used to disseminate key messages about good dog care (social media is widely used in Sanur, although mostly actively by younger people). But it takes time to craft and approve content and the numbers of likes and/or followers are still relatively low.
6. It can be difficult to definitively identify an unowned in the database.
7. It takes a long time to establish trust, active engagement and in-kind support from local authorities.
8. Difficult to face complaints from community members who don't like dogs or have unrealistic expectations of solving issues with dogs.

## Discussion

This study reports the results of an evaluation, combining quantitative data analysis and participatory exercises to emphasize lessons learnt from 2 years of implementing a novel community-based dog welfare program for rabies control, named Program Dharma. Although every effort was made to identify all dogs living in the three *desas*, the sample of dogs used in the analysis may have been a biased sample, as these are the dogs owned by people most engaged in Program Dharma as they have been revisited within the past year (between June 2017 and June 2018), other dogs living in the same community but not revisited by a Program Dharma representative may not have shown such improvements. Hence our study tests whether engagement with a community-based program improves welfare and reduces rabies risk, rather than can such a program change welfare and rabies risk across a whole community, regardless of the level of individual owner engagement. Particularly for rabies risk, where vaccination must be comprehensive throughout the population for effective herd immunity (3), the effect of Program Dharma on all dogs in the community regardless of frequency of direct program contact is important and will be the subject of our future studies.

The data collection method for dog demography, health and welfare was designed to create repeated measures for each dog and to incorporate data collection into the daily work of T2s. This method required identifying the dog in the existing database during revisits. The dogs had not been individually marked, instead a combination of a photo, physical description, dog name plus owner name and address was expected to be sufficient. However, identification proved challenging. Some of the dogs and their families had moved out of the desa, or a dog that had died/been given away had then been forgotten by the family members present at revisit. However, the majority of identification challenges appear to be due to data collection errors during the baseline which were difficult to rectify as personnel changed between baseline and revisits. These errors include insufficient description of household location, recording wrong dog name (or a name that is rarely used and not recognized by all family members) combined with poor quality photo for dog recognition and recording the name of the interviewee as opposed to the head of household. As households are commonly comprised of extended families the interviewee name may be different to the main recognized family name for the household. These errors made identifying either the house or dog difficult. If there was a dog present in the household, but not identified in the database, it would be entered as a new dog. Hence some of the original census dogs were retained in the sample, although with a new later baseline, the number of these reentered dogs was not possible to calculate. These errors were noted early in the project and rectified as data collection protocols were refined. Permeant individual identification would have helped with finding dogs in the database. Alternatively, separate from the regular T2 visits, a specific data collection effort resulting in a repeated census or cross-sectional sample could have been used to monitor changes in the dog population.

The evaluation event relied on analysis and interpretation of objective data but also on the subjective assessments of community representatives. During the evaluation event, the T2 groups were facilitated by their T1 village coordinator, this may have biased responses as village coordinators had a supervisory role with T2s. T2s and T1s had a close and positive working relationship, often visiting dogs and owners together, and appeared to use their shared experiences of Program Dharma activities to develop responses in a collaborative and non-hierarchical way. However, it would have been ideal to use facilitators without supervisory responsibility to avoid any potential bias. Further, as these representatives were engaged in Program Dharma activities themselves, they may have been biased toward seeing positive outcomes of Program Dharma. Future evaluations should look to include the perspectives of community members outside the Program Dharma team and should explore the vaccination status and welfare of dogs owned by people not re-visited by the program.

Vaccination services were principally provided by local government (Dinas Pertanian Kota Denpasar). All *desas* showed an increase in current vaccination coverage during Program Dharma activity; however this increase was only statistically significant in two *desas* and not in Kelurahan Sanur. In Kelurahan Sanur there was a decrease in unvaccinated dogs but also a substantial concurrent increase in dogs with lapsed vaccination (vaccinated over 12 months previously), leading to the proportion of dogs with current vaccination status showing a statistically insignificant increase. Prior to Program Dharma, mass vaccination was delivered primarily through a central point strategy. A proportion of owners brought their dogs every year, leading to a low proportion of lapsed vaccinations. However, Program Dharma identified a population of dogs that had never been vaccinated via this central point approach. T2s were able to indicate to local government vaccinators where these unvaccinated dogs could be found allowing subsequent mass vaccination campaigns to use a targeted door-to-door strategy to reach them. Limited vaccine meant this reduced some central point efforts resulting in an increase in lapsed vaccination for those dogs that had relied on central point services. This appears to have been particularly pronounced in Kelurahan Sanur. However, the mass vaccination campaign uses as vaccine providing 3 years of immunity (Rabisin®) so a lapsed vaccination does not necessarily mean susceptibility to rabies. This approach appears to have prevented any rabies virus incursions as there were no reported dog or human cases of rabies during Program Dharma activity.

Predictors for rabies vaccination uptake in other studies in Indonesia have included dog owner socio-economic status, knowledge of vaccination campaigns, ability to handle dogs and characteristics of the dogs themselves including age and level of confinement (4, 21–24). However, the three *desas* in our study are extremely similar in terms of these predictors. Reasons for the less substantial increase in current vaccination and greater increase in lapsed vaccination in Kelurahan Sanur may be more to do with differences in how vaccine has being delivered during the annual mass vaccination campaigns than dog or owner characteristics.

In all three *desas*, comparing dogs at baseline to their condition at the most recent visit by a Program Dharma, revealed a large and statistically significant reduction in the proportion of dogs in poor welfare (poor welfare defined as thin or emaciated body condition, a visible skin problem or a visible injury). This improvement in welfare is proposed to be due to an improvement in care provided by owners following encouragement by Program Dharma and/or access to veterinary care provided through Program Dharma “Health Days.”

Over the period of Program Dharma activity, we observed a change in confinement practices with an increase in confinement of dogs within the household (these are dogs that are allowed access to the house and are prevented from roaming by a wall/fence that surrounds the household including the yard) and a decrease in all other methods of confinement. The reduction in kenneling/caging and tethering was desired as this is extreme behavioral restriction for a dog and hence has welfare implications. The reduction in free-roaming was desired as this would reduce the risk of road traffic accidents, feces in public places and potential nuisance for community members. However, dogs that have transitioned from free-roaming to caging or tethering are indicated with red cells in the transition matrix as there is a potential welfare concern for these dogs as they have moved from having relatively free movement to extreme confinement. Where confinement is to be encouraged, this must be done humanely to avoid both dog welfare concerns and potentially creating dog behavior problems for owners caused by a sudden increase in confinement.

Early morning street surveys provided a measure the density of roaming dogs, expressed as the number of dogs per km of street surveyed; the densities observed in the 3 *desas* fell within the range of densities observed in seven locations reported in Hiby and Hiby (20). There was a significant reduction in the density of roaming dogs over time across all three *desas*. As is common in many developing world locations, the majority of dogs seen roaming on public streets in Bali are owned dogs allowed to roam without supervision by their owners (1, 10, 22). Hence this observed reduction in roaming dog density is likely influenced by the increase in confinement practices by owners during the period of Program Dharma activity. Theoretically, an increase in sterilization would lead to a reduction in roaming, principally because there would be fewer females in heat, attracting males to congregate around them. However, our data only show a significant increase in sterilization of owned dogs in one *desa*, hence our data do not support this is the primary cause for the observed reduction in roaming. Sterilization may still be playing a role in reducing roaming dog density, as unowned dogs were prioritized for the 621 sterilizations conducted via “Health Days.” However, the more significant changes in vaccination status and improved visible welfare suggest improved care giving by owners as an alternative explanation. Dogs roam for many reasons but foraging for food is one proposed motivation that will have been reduced by owners providing more or better quality food at home, which would have similarly contributed to the observed improvement in body condition. Further exploration into the factors contributing to changes in confinement status is warranted. A final potential contributing factor to a fall in roaming density may have been a reduction in abandonment, a strongly desired impact of Program Dharma due to the welfare risks of abandoned dogs and challenges in maintaining current vaccination without a responsible owner. Program Dharma encouraged owners to keep dogs for life by changing care practices to mitigate unwanted dog behaviors, accessing veterinary treatment for sick dogs rather than abandon them and sterilizing female dogs whose puppies would be unwanted. Measuring change in the rate of abandonment is difficult as owners rarely openly admit such practice and there is anecdotal evidence that owners from other areas abandon their dogs in Sanur. There was a reduction in the proportion of unowned dogs revisited in the most recent 12 months of Program Dharma activity, which could indicate a reduction in abandonment, however conclusively identifying unowned dogs is challenging. These roaming dogs may be owned dogs that have traveled over the border from another *banjar* and are hence not on the *banjar*–specific “Dogalog” catalog held by the T2. As revisit data is only submitted when dogs are clearly recognized and matched to their “Dogalog” entry, the apparent reduction in unowned dogs in the revisit dataset may reflect these identification difficulties rather than an actual reduction in unowned dogs in the community.

We note that our desire to see a reduction in the density of roaming dogs through Program Dharma is for reasons of reducing the risks to dog welfare and public health through road traffic accidents, feces in public places and negative interactions between roaming dogs and people which can include dog bites. However, it's important to note that there is no evidence that a reduction in roaming dog population density has any impact on rabies transmission, as this is density independent (7, 23). One hypothesis for this density independence is that a clinically rabid dog can move long distances until prevented from traveling any further by people or dogs, or progression of the disease making the dog effectively immobile. If prevented from traveling further through fighting with a dog that is vaccinated, the transmission chain will end with that vaccinated dog as its immune system will kill any virus transmitted during the fight. Hence Program Dharma's rabies related goal was to increase vaccination rather than reduce roaming.

The evaluation also revealed lessons about program implementation. Positive learnings include:

- Using local representatives from the community appears to be a benefit, as compared to “outsiders” who are not immediately trusted. The very low rate of owner refusal to participate in interviews, presumably helped by T2s being recognized local community members, is an example of this benefit.
- Community members appeared to respond well to T2s, welcoming them into their homes to discuss their dogs, inviting the team to community events and providing funds from community resources to cover some Program Dharma costs.
- The Program Dharma catalog (aka “Dogalog”) designed to track changes in dog care and welfare was also useful for identifying where unvaccinated dogs live for targeting during government mass vaccination; and has on occasion allowed reuniting of lost dogs.

Implementation challenges include:

- A large portion (45%) of the baseline dataset could not be identified during revisits.
- Sustaining the benefits created through Program Dharma is economically most challenging due to the costs of veterinary care provided through “Health Days”; there is a risk of creating owner dependency upon free veterinary care through the program.
- T2s were paid for the time they spent on Program Dharma, this adds another challenge to sustaining the program in the long term, although there has been some progress in addressing this challenge, with two of the three *desas* contributing some local funding to cover this cost.
- Provision of vaccination services is currently restricted to local government vets, Program Dharma veterinary staff were not permitted to vaccinate dogs. This reduced vaccination opportunities and hence coverage was lower than it could have been.

This first evaluation of a novel community-based approach to dog welfare and rabies control reports several beneficial impacts for vaccination coverage, dog care and welfare; as well as challenges to implementation. The benefits were judged to be sufficient to evolve and extend Program Dharma to new areas. Although this evaluation focused on a sample of dogs that had been recently revisited by T2s, future evaluations will have the opportunity to explore Program Dharma impacts in the wider community.

## Data Availability

The datasets for this manuscript are not publicly available because they contain dog owner personal details required by T2s to acomplish revisits. Requests to access the datasets should be directed to Elly Hiby, ellyhiby@gmail.com.

## Ethics Statement

The research aspects of Program Dharma underwent ethical review and received ethical clearance, No 356KE-PH-Lit-3II2018, in the Faculty of Veterinary Medicine, Udayana University, Bali. All data collection was conducted in accordance with the Declaration of Helsinki, participation was voluntary, following prior informed consent.

## Author Contributions

KNA, EH, JG, NU, IMS, SP, IBNS, KKA, PJ, and DW: conceptualization. KNA, EH, JG, NU, IMS, SP, IBNS, KKA, and DW: methodology. EH: software. EH, LH, and MH: formal analysis. JG, YH, LK, GB, HI, NU, and KKA: investigation. NU and EH: writing—original draft preparation. IMS, SP, IBNS, KKA, PJ, IMIS, and DW: writing—review and editing. All authors contributed to approving the submitted version. KNA: funding acquisition.

### Conflict of Interest Statement

LH was employed by company Conservation Research Ltd and declares no competing interests. The remaining authors declare that the research was conducted in the absence of any commercial or financial relationships that could be construed as a potential conflict of interest.

## Footnotes

^1^https://www.cdc.gov/epiinfo

^2^https://www.devicemagic.com

^3^http://www.wisemonkeyfoundation.org

## References

[1] Mustiana A Toribio J-A Abdurrahman M Suadnya IW Hernandez-Jover M Putra AAG. Owned and unowned dog population estimation, dog management and dog bites to inform rabies prevention and response on lombok island, Indonesia. PLoS ONE. (2015) 10:e0124092. 10.1371/journal.pone.0124092 25932916 PMC4416720

[2] Putra AAG Hampson K Girardi J Hiby E Knobel D Mardiana IW. Response to a rabies epidemic, Bali, Indonesia, 2008-2011. Emerg Infect Dis. (2013) 19:648–51. 10.3201/eid1904.120380 23632033 PMC3647408

[3] Townsend SE Sumantra IP Pudjiatmoko BGN Brum E Cleaveland S Crafter S. Designing programs for eliminating canine rabies from islands: Bali, Indonesia as a case study. PLoS Negl Trop Dis. (2013) 7:e2372. 10.1371/journal.pntd.0002372 23991233 PMC3749988

[4] Arief RA Hampson K Jatikusumah A Widyastuti MD Basri C Putra AA. Determinants of vaccination coverage and consequences for rabies control in Bali, Indonesia. Front Vet Sci. (2016) 3:123. 10.3389/fvets.2016.00123 28119919 PMC5220097

[5] Putra AAG Special efforts to accelerate eradication of rabies in Bali Province. In: Upaya Percepatan Pemberantas Rabies Di Provinsi Bali / Efforts to Accelerate Eradication of Rabies in Bali Province. Denpasar, (2019).

[6] Mardiana IW Activities for controlling and eradication of rabies in Bali Province. In: Workshop on Coordinating Rabies Eradication in Bali Province. Denpasar, (2018).

[7] Hampson K Dushoff J Cleaveland S Haydon DT Kaare M Packer C. Transmission dynamics and prospects for the elimination of canine rabies. PLoS Biol. (2009) 7:e1000053. 10.1371/journal.pbio.1000053 19278295 PMC2653555

[8] Vigilato AMN Clavijo A Knobl T Silva HMT Cosivi O Schneider MC. Progress towards eliminating canine rabies: policies and perspectives from Latin America and the Caribbean. Phil Trans R Soc B. (2013) 368:20120. 10.1098/rstb.2012.0143 23798691 PMC3720041

[9] Coleman PG Dye C Short Papers Immunization coverage required to prevent outbreaks of dog rabies. Vaccine. (1996) 14:185–6. 10.1016/0264-410X(95)00197-9 8920697

[10] Hiby E Karang K Id A Atema KN Bagus GN Girardi J Dog ecology and rabies knowledge of owners and non-owners in sanur, a sub-distrcit of the indonesian island province of bali. Animals. (2018) 8:1–18. 10.3390/ani8070112 PMC6070915 29976915

[11] Cleaveland S Kaare M Tiringa P Mlengeya T Barrat J. A dog rabies vaccination campaign in rural Africa: impact on the incidence of dog rabies and human dog-bite injuries. Vaccine. (2003) 21:1965–73. 10.1016/S0264-410X(02)00778-8 12706685

[12] Lembo T Hampson K Kaare MT Ernest E Knobel D Kazwala RR. The feasibility of canine rabies elimination in Africa: dispelling doubts with data. PLoS Negl Trop Dis. (2010) 4:e626. 10.1371/journal.pntd.0000626 20186330 PMC2826407

[13] Reece JF Chawla SK. Control of rabies in Jaipur, India, by the sterilisation and vaccination of neighbourhood dogs. Vet Rec. (2006) 159:379–83. 10.1136/vr.159.12.379 16980523

[14] Zinsstag J Lechenne M Laager M Mindekem R Naïssengar S Oussiguéré A. Vaccination of dogs in an African city interrupts rabies transmission and reduces human exposure. Sci Transl Med. (2017) 9:aaf6984. 10.1126/scitranslmed.aaf6984 29263230

[15] Azhar M Lubis AAS Siregar AES Alders ARG Brum BE Mcgrane BCJ. Participatory disease surveillance and response in indonesia: strengthening veterinary services and empowering communities to prevent and control highly pathogenic avian influenza. Avian Dis. (2010) 54:749–53. 10.1637/8713-031809-Reg.1 20521726

[16] Berrian AM Smith MH Rooyen J Van Martínez-lópez B Plank MN Smith WA. A community-based One Health education program for disease risk mitigation at the human-animal interface. One Heal. (2018) 5:9–20. 10.1016/j.onehlt.2017.11.002 29270459 PMC5734692

[17] Cyril S Smith BJ Possamai-inesedy A Renzaho AMN. Exploring the role of community engagement in improving the health of disadvantaged populations: a systematic review. Glob Health Action. (2015) 8:29842. 10.3402/gha.v8.29842 26689460 PMC4685976

[18] World Health Organisation Key Messages for Social Mobilization and Community Engagement in Intense Transmission Areas. (2014). Available online at: https://www.who.int/csr/resources/publications/ebola/social-mobilization-guidance/en/

[19] Nugroho DK Pudjiatmoko IKD Tum S Schoonman L Analysis of rabies surveillance data (2008-2011) in Bali Province, Indonesia. Outbreak Surveill Investig Rep. (2013) 6:8–12. Available online at: http://www.osirjournal.net/index.php/osir/article/view/51

[20] Hiby E Hiby L. Direct observation of dog density and composition during street counts as a resource efficient method of measuring variation in roaming dog populations over time and between locations. Animals. (2017) 7, 57. 10.3390/ani7080057 28771177 PMC5575569

[21] Wera E Mourits MCM Hogeveen H. Intention of dog owners to participate in rabies control measures in Flores Island, Indonesia. Prev Vet Med. (2016) 126:138–50. 10.1016/j.prevetmed.2016.01.029 26898353

[22] Morters MK Bharadwaj S Whay HR Cleaveland S Damriyasa IM Wood JLN. Participatory methods for the assessment of the ownership status of free-roaming dogs in Bali, Indonesia, for disease control and animal welfare. Prev Vet Med. (2014) 116:203–8. 10.1016/j.prevetmed.2014.04.012 24910425

[23] Morters MK Restif O Hampson K Cleaveland S Wood JLN Conlan AJK. Evidence-based control of canine rabies: a critical review of population density reduction. J Anim Ecol. (2013) 82:6–14. 10.1111/j.1365-2656.2012.02033.x 23004351 PMC3579231

[24] Wera E Mourits MCM Hogeveen H. Uptake of rabies control measures by dog owners in flores Island, Indonesia. PLoS Negl Trop Dis. (2015) 9:1–23. 10.1371/journal.pntd.0003589 25782019 PMC4363700

