# Supplementary material for: Evaluation of Community-Based Dog Welfare and Rabies Project in Sanur, a Sub-district of the Indonesian Island Province of Bali
Source: Front Vet Sci. 2019 Jul 9;6:193. doi: 10.3389/fvets.2019.00193 (PMC6629783; doi:10.3389/fvets.2019.00193)
Supplement: Supplementary file 1 [file Table_1.DOCX]

Supplementary Material

# Participatory community-based agent (T2) evaluation event: Facilitation notes

# Facilitation team:

- Lead facilitator to introduce exercises and facilitate plenary
- Three working group facilitators; Mentors (T1) village coordinators to lead each of their three T2 village working groups
- One-two T1 “listeners” to be assigned to each village working group to capture specific Most Significant Change stories that arise

# Objectives:

- Recognise and celebrate T2 contribution to Program Dharma (PD)
- Project team understands how T2s see their role and how they organize their work
- Establish positive and negative impacts of PD on different beneficiaries from T2 perspective
- Identify ways to improve PD training and implementation for maximum effectiveness

# Outline of day:

1. Thank you to the T2s for their work:
   - We recognize and respect the work you are doing to make PD a success. The goals of this day are to hear from you about the successes and challenges of PD from T2 perspective, and to make sure your voice is heard as we assess the success of the program. At the end of this session, we would like to thank you for your participation with a small celebration.
2. Review Agenda
3. Consent statement – read out and ask for verbal agreement and then signature on a print out of the statement
4. Warm up exercise (only if time allows)
5. Participatory exercises:
   - T2 tasks and time allocation
   - T2 successes and challenges
   - Factors that contribute to successes and challenges
   - Circles of impact: Dogs, Dog owners, *Banjar*, Village and Municipal leadership
6. Thank you celebration and lunch

# Participatory Exercises

## T2 Tasks and time allocation

Break into *desa* groups

***Step 1***

- **(Lead facilitator)** Instruct T2s to spend 5-10 minutes to think about every task they perform as part of Program Dharma.
- **(T1 facilitators)** As T2s list tasks, facilitator (and/or listeners) can assist by writing ONE TASK PER STICKY clearly
- If T2 says something that is a group of tasks, like “collect data”, acknowledge the contribution and prompt them to break it up, such as: “Ok, data collection. What are the different tasks involved in data collection? One thing is to update the “Dogalog” on the tablet, right? What comes next?”
- Record each different task on one sticky. It is ok if there are duplicates.
- When they can’t think of any more, put all the stickies on the wall (flipchart) and read them aloud. Ask if there are any other things they do as part of Program Dharma. For instance, do they sometimes spend time answer questions, even when they aren’t formally working?
- List any new ideas, until everyone confirms that the list is complete. If there are duplicates, put them together, confirming with the group that they are actually the same activity (e.g. data collection in the “Dogalog” and “collecting data during door to door visits”).

***Step 2***

- **(Lead facilitator)** Now, ask the T2s to think about how much time they spend on each task in AN AVERAGE MONTH. Not a month when tablets are missing, or many ceremonies, but a normal month. How much time is spent on each activity in total? Clarify, not ONE door to door visit, but TOTAL TIME SPENT on door to door visits in a normal month.
- Ask T2s to arrange the “task” stickies from left to right, least to most time spent.
- **(T1 facilitators)** Assist with the task, ensuring everyone understands the task and is in agreement with the group’s decisions. Move the stickies for them if necessary, by clarifying: “In a normal month, do you spend more time on [task A] or [task B]?”
- **(Lead facilitator):** Thank them for their work, note that we will take pictures of the exercises, and direct them to the next task.

## Successes and Challenges

Staying in *desa* groups

### Successes

***Step 1***

- **(Lead facilitator)**: Now we will ask you to think about the successes and the challenges of Program Dharma, from your own perspective. With the T1s in your *desa,* let’s first think about the successes of Program Dharma. What are the best things about Program Dharma, from your perspective?
- **(T1)** Direct the group to pre-prepared poster paper, with + on top, and – on the bottom. Starting with successes, list ONE SUCCESS/GOOD THING PER STICKY. As you write it, read it aloud again and place it on the poster in the + section. Duplicate or similar answers are ok and should ALL be listed, but similar answers may be grouped near each other on the poster.
- ***(Listeners)*** Your role is important here! Listen for key stories, note who made each comment, and follow up with them later to get a statement or the longer story. Do NOT interrupt the flow of the exercise, just make notes.
- **(T1)** Ensure everyone knows that they can list as many successes, or good things, as they can think of.
- When everyone is done, read all the successes aloud, clarify that they have been captured accurately, and wait for Lead facilitator.

***Step 2***

- **(Lead facilitator):** Now the we have listed our successes, let’s think about what contributes to those successes. For instance, if you said “dogs are healthier”, why is that? Maybe you want to write “Health Days” or “more attentive owners”.
- For each success or group of successes, use a different color sticky to tell us what you think makes that success possible.
- **(T1)** Direct group to each success (or group of successes, if they are grouped). On a DIFFERENT COLOR, list one “helpful factor” per sticky, and place them near the successes they support. Go around until each success (or group of successes) has been addressed. It is ok to list the same factor for different successes.
- Confirm that the group has talked about all the things in Program Dharma that contribute to their successes

### Challenges

***Step 3***

- **(Lead facilitator):** Now we will talk about the challenges you face when implementing Program Dharma. What is difficult for you, or not going so well?
- **(T1 facilitator):** Using a NEW COLOR, ask the group to tell you about challenges or problems, listing ONE PER STICKY. Just like above, duplicates are ok, but place them in groups if they are very similar.
- Confirm that the group has listed all their challenges.

***Step 4***

- **(Lead facilitator):** Direct the group to now think about what contributes to those challenges, or what in Program Dharma could change to fix that challenge. For instance, if you said, “People don’t come to Health Days” you might say, as a contributing factor “Health Days happen while they are at work, or ‘Change the time of Health Days so everyone can come’”. Either is fine.
- **(T1 facilitator)** Help group address each challenge, by asking what are the things that either make that issue problematic, or what PD could change to fix the problem.
- Avoid discussing issues outside the control of PD, if possible, such as “People shouldn’t be allowed to own dogs”. Try to focus on PD-related factors or potential changes to help improve things.
- **(Lead facilitator):** Thank everyone, and give the groups five minutes to look at each other’s charts, if they would like. Do NOT go change your own after seeing others’ though. We want each chart to represent your own ideas first.

## Circles of Impact

Staying in *desa* groups

***Step 1***

- **(Lead facilitator)** Now we will work with our *desa* groups, but all contribute to the same chart. You will be asked to think about the positive and negative impacts of Program Dharma on different groups. Here (point to chart), we ask about the positive and negative impacts of PD on DOGS in your *banjar*. Here (point) we ask about the positive and negative impacts on DOG OWNERS. This chart is for impacts on YOUR *BANJAR*, and that one (point) is for positive and negative impacts on *DESA* AND MUNICIPAL LEADERSHIP.
- Each group with start with a different chart. Put yourself in the perspective of that group. Think about all the ways PD impacts that group, placing positive impacts on the top, and negative impacts on the bottom. If you can’t decide if it is positive or negative, it can go somewhere in the middle.
- After you finish the first one, I’ll give you instructions for the next one.
- **(T1 facilitator):** Help your group think about impacts for the group they have chosen. ONE IMPACT per sticky.
- Help ensure that all ideas are written down.
- Ask the group if it’s a positive or a negative impact. Ask them to place it in the circle, with positive impacts on top, and negative on the bottom. If they can’t decide, place it somewhere near the middle.
- WAIT until Lead facilitator gives the next instruction. DO NOT move to the next circle.

***Step 2-4***

- **(Lead facilitator)** Now you will move to the next impact circle. Here, first review when the group before you has written. If you agree with that statement, place one circle, with your *desa’s* color, on that statement.
- Then, if you have anything new to add, please add that to the circle.
- **(T1):** Make sure that the group follows instructions, does not write duplicate responses (but it’s ok if there are similar responses), and ensure that all ideas from the group are recorded.
- **(Listeners):** Capture any interesting stories so you can follow up!

***Step 5***

- **(Lead facilitator)** Ask T1 facilitators to hand 5 stickers to every participant. Instruct participants to review ALL the impact circles. They get 5 votes – one per sticker. They should place those votes on the impacts they believe are the MOST important. They can be positive or negative impacts, and they are allowed to place more than one sticker per impact if they believe it to be especially important. They have 5 minutes to review and place all their sticker “votes”.
- Review the impacts that received the highest impact, and celebrate the many important impacts that PD is having on their communities, with the important work of the T2s.
- Congratulate everyone for their work, and thank them for their participation. Tell them we’d like to thank them with a small celebration, followed by lunch if they would like to stay.
